# Maturation of Airway Defensive Reflexes Is Related to Development of Feeding Behavior during Growth in Rabbits

**DOI:** 10.3389/fphys.2017.00064

**Published:** 2017-02-08

**Authors:** Laurianne Coutier-Marie, Iulia Ioan, Claude Bonabel, Bruno Demoulin, Anne-Laure Leblanc, Ludivine Debitu, Cyril Schweitzer, François Marchal, Silvia Demoulin-Alexikova

**Affiliations:** ^1^EA 3450DevAH – Laboratoire de Physiologie, Faculté de Médicine, Université de LorraineVandœuvre-lès-Nancy, France; ^2^Service d'Explorations Fonctionnelles Pédiatriques, Hôpital d'EnfantsVandœuvre-lès-Nancy, France; ^3^Service de Pneumologie, Hôpital Femme-Mère-Enfant, Hospices Civils de LyonLyon, France

**Keywords:** rabbit pups, mechanical stimulation, tracheal stimulation, defensive airway reflex, growth, swallowing, expiration reflex, cough

## Abstract

**Introduction:** Cough and expiration reflex are major lower airway defense mechanisms that have not been studied throughout development in relation with the feeding behavior.

**Aim:** To describe airway defense reflexes evoked by mechanical stimulation of the trachea in developing rabbit pups.

**Material and Methods:** Sixty one pups were allocated to 3 groups according to their feeding behavior: suckling (*n* = 22), weanling (*n* = 21) and weaning (*n* = 18) group. The incidence and sensitivity of defense reflexes triggered by mechanical tracheal stimulation were studied in anesthetized and tracheotomized animals. Data are expressed as median (25th to 75th percentile).

**Results:** The overall incidence of defensive responses (cough and/or expiration reflex) was found to be significantly higher in suckling [100% (50–100%); *p* = 0.01] and weanling [75% (40–100%); *p* = 0.05] animals when compared to weaning ones [37.5% (0–75%)]. However, cough motor pattern accounted for only 29% (0–62%) of all defensive responses in suckling rabbits and its frequency was significantly lower in this group when compared with weanling [100%(50–100%); *p* = 0.006] or weaning group [62%(50–100%), *p* = 0.05]. In other word the expiration reflex was the dominant response in suckling animals.

**Conclusion:** Incidence and motor pattern of defensive responses were found to be linked to the pup feeding behavior and the expiration reflex was the major response triggered in suckling pups. The results suggest that this reflex is especially fitted to occur during the coordinated swallowing - breathing fast activities of sucking.

## Introduction

Important pediatric specificities of cough diagnosis and management have been pointed out, as a result of etiological factors, pathophysiological mechanisms and therapeutic issues (Chang, 2005). Cough is most frequent in children where it may accompany/reveal a variety of respiratory conditions. The prevalence of chronic cough is 5 to 10 % at preschool age (Chang, 2005; Chang and Widdicombe, 2007) and acute cough is a common symptom of respiratory tract infection. It is reported in infants with respiratory syncytial virus bronchiolitis, although those less than 2 months may rather be prone to apnea (Ogra, 2004; Ravaglia and Poletti, 2014). Apnea also is the worrisome response reported at intra-partum/post-delivery oro-naso-pharyngeal suctioning (Velaphi and Vidyasagar, 2008).

On the other hand, there is some evidence that active airway defense mechanisms are present during early development. For instance, spontaneous cough has been reported in a non-negligible proportion of unselected newborns in the delivery room (Miller et al., 1952). That the sound associated with cough may actually encompass 2 types of expulsive response is usually not taken into account in most descriptions during development. The expiration reflex is a brief expiratory effort, while cough is a complex response that requires the precise space - time coordination of nearly all respiratory muscles (Widdicombe and Fontana, 2006; Poliacek et al., 2008; Tatar et al., 2008). We are aware of little detailed, systematic assessment of each type of response throughout growth, except for the early experimental documentation of the expiration reflex at birth (Korpas and Tomori, 1979).

The aim of the study was therefore to assess airway defense reflexes, during growth and development, in an animal model that was designed to test ventilatory responses to mechanical stimulation of the trachea (Varechova et al., 2010). The model was adapted to study the occurrence of these reflexes at different stages of development, defined from the feeding behavior. The staging was adopted because of the critical importance of coordinating breathing and swallowing to protect the airways with specific mechanisms at this period of life (Lau, 2015). The hypothesis was that the dual nature of airway defensive reflexes would account for the characteristics of these responses during development.

## Materials and methods

### Animals

Animal experiments were carried out in 2012, and in conformity with the European Communities Council (ECC) guidelines for animal care procedures and the French legislation application of the ECC directive 86/609/EEC (decree number 87-848). The study was conducted under the license (A54518-03409) from the French ministry of agriculture and fisheries (*Ministère de l'Agriculture et de la Pêche*) and the Ministry of higher education and research (*Ministère del'EnseignementSupérieur et de la Recherche*; A54518-03409) and supervision by the regional veterinary services (*Services Vétérinaires Départementaux de Meurthe et Moselle*). Ethics committee approval was not required, in accordance with French legislation in place at the time of the study.

Seventy seven rabbit pups had been inbred for the study and were randomly allocated to 3 experimental groups according to feeding behavior. The experimental protocol was performed in the 1st group while less than 21 days old (suckling); in the 2nd group while 21–40 days old (weanling) and in the 3rd group while aged more than 40 days (weaning). The study was completed in a total of 61 rabbits; 16 rabbits were excluded because of inter-current respiratory infection or death during anesthesia induction or during the experiment.

### Anesthesia, analgesia, and euthanasia

The induction of surgical anesthesia was performed by intraperitoneal administration of pentobarbital (Pentobarbital® sodique, Ceva Santé Animale, France) in rabbits less 21 days old and by intravenous administration of pentobarbital through the marginal ear vein in rabbits aged more than 21 days. The dose of pentobarbital was 15 mg/kg in rabbits less than or equal to 15 days old and 30 mg/kg in rabbits aged more than 15 days. Pre-operative local analgesia before tracheotomy was performed by subcutaneous administration of Laocaine® 20 mg/ml (Intervet, Schering-Plough Animal Health, France). After induction, the depth of anesthesia and analgesia was assessed every 20 min by testing the pedal withdrawal reflex (foot pad pinch on both hind feet) and respiratory rate. In the case of the animal responsiveness to painful stimuli and/or increase in respiratory rate, the animal was supplied with additional anesthesia and re-tested before continuing experimental procedures.

Euthanasia was achieved with intravenous administration of Doléthal® (Vétoquinol S.A., France) in a dose of 100 mg/kg for rabbits less than 15 days and 200 mg/kg for older ones.

### Animal preparation

The anesthetized animal was placed in the supine position on a heating pad to maintain body temperature between 38 and 39°C and left to rest for20 min. The electromyogram of the transverse abdominal muscle was performed by insertion of bipolar insulated fine-wire electrodes according to Basmajian and Stecko (1963) to further characterize the active expiration of cough and expiration reflex. An upper cervical tracheotomy allowed the insertion of a tracheal cannula that was connected to a pneumotachograph and to the mechanical stimulation apparatus. A No. 0 Fleisch pneumotachograph (linear range ± 250 mL/s) measured flow in rabbits older than 40 days and was calibrated before each experiment using a 20 ml calibration syringe. A custom-made flowmeter was developed in the laboratory with a linear range ± 40mL/s to measure flow in pups aged less than 40 days. The homemade flowmeter was tested for linearity with Fleisch Pneumotachograph No. 00 and was calibrated before each experiment using a 10 ml calibration syringe.

### Mechanical stimulation of the trachea

The mechanical stimulation of the trachea (at the level of the carina) was performed using a semi-rigid wire (nylon fiber) rotated by a23HSX-102 universal motor (Rare Earth Magnet Stepper Motors ref. 23HSX-102, Mclennan Servo Supplies Ltd., Ash Vale, UK) that achieved a square wave stimulus automatically triggered 50 ms from the beginning of inspiration. The beginning of inspiration was detected electronically as soon as the flow signal reached a positive value.

**Figure 1:**
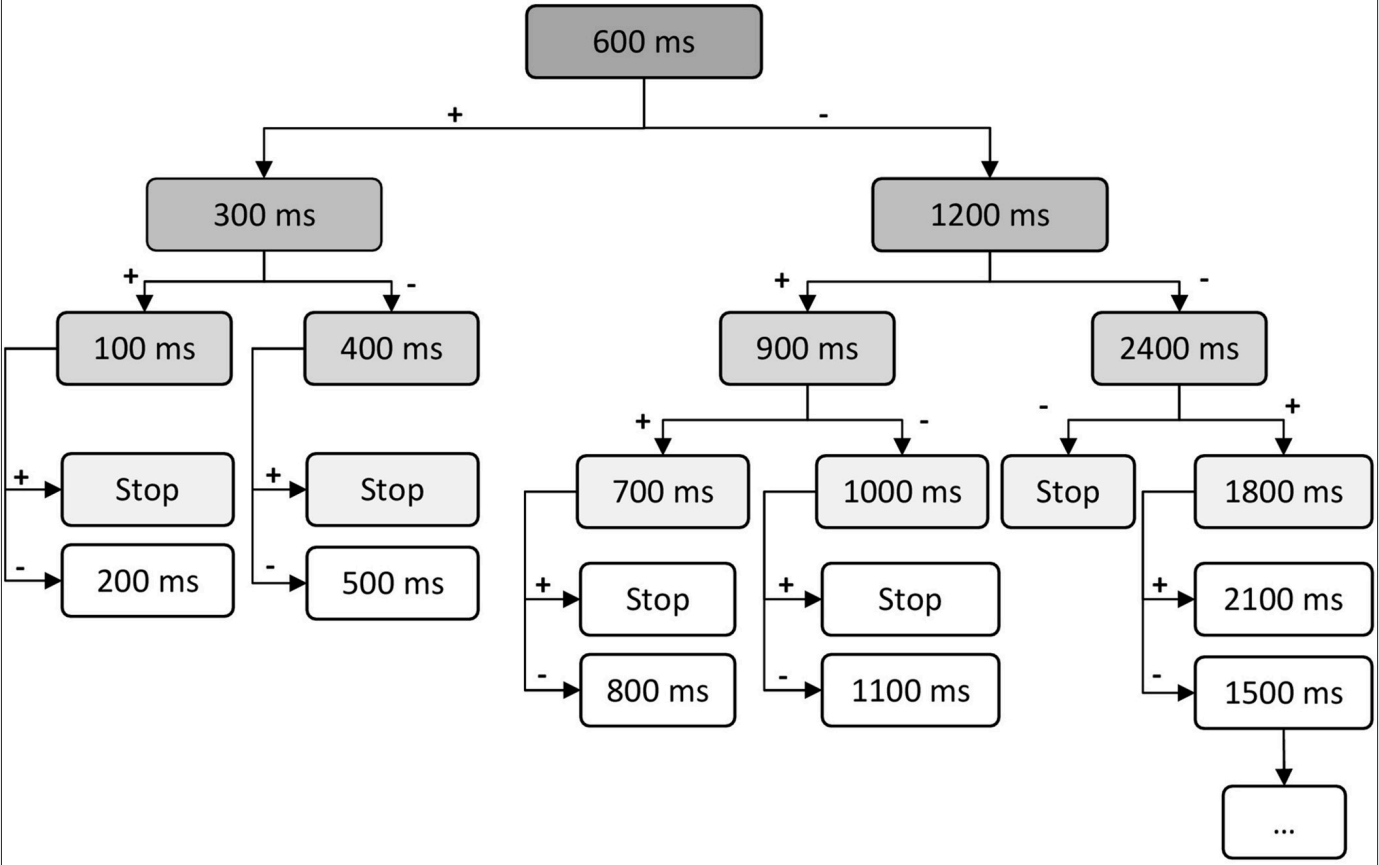
**Decision diagram of tracheal stimulation**. Tracheal stimulation protocol starts with the standard 600 ms stimulus. Each animal underwent 8 stimulations with the initial 600 ms stimulus. Duration of stimulus is decreased or increased stepwise, depending respectively on occurrence (+) or absence (−) of defensive response. Each stimulation step using stimulation duration other than 600 ms was tested 4 times.

The stimulation time could be varied in a stepwise manner between 100 and 2400 ms. Stimulation was performed when breathing was regular. The initial stimulus was set to 600 ms, a duration that has been shown to readily elicit a response in mature rabbits (Varechova et al., 2011). Each animal underwent 8 stimulations with the initial 600 ms stimulus. Depending on the response observed, the stimulation duration was altered stepwise - down to 100 ms or up to 2400 ms (according to the protocol detailed in Figure 1) in order to identify a threshold for the shortest stimulus able to elicit a response. Each stimulation step using stimulation duration other than 600 ms was tested 4 times. At least 1 min was allowed to elapse between two mechanical stimulations. The signals of airflow, integrated volume and stimulus were fed to the LabChart recorder (ADinstruments, Oxford, UK), digitized at 1000 Hz and used for on-line and off-line analysis.

### Ventilatory responses to tracheal stimulation

#### Types of defensive responses

**Figure 2:**
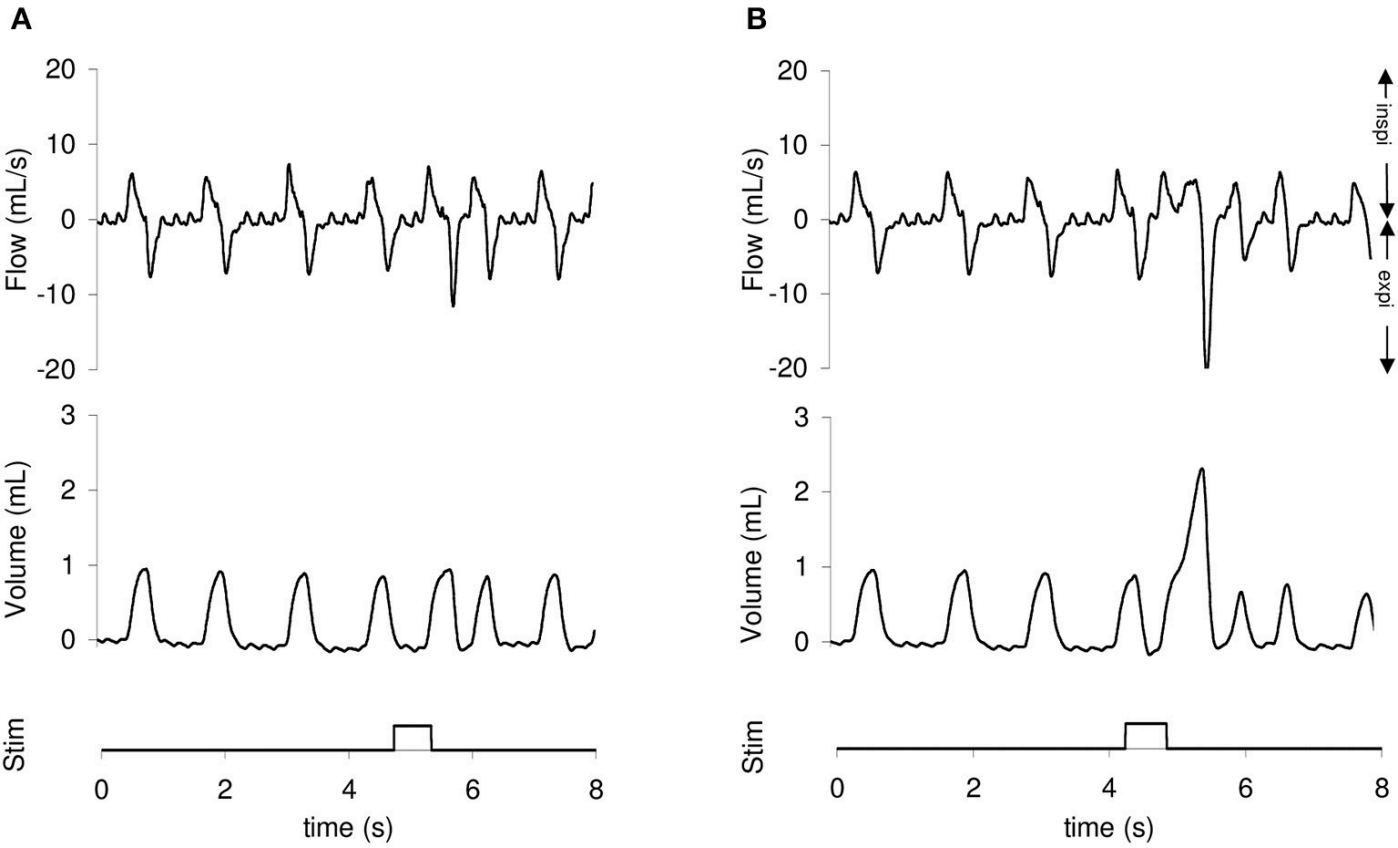
**Airflow and tidal volume recordings illustrating occurrence of expiration reflex (A)** and cough reflex **(B)** triggered by a mechanical stimulation of the trachea in a 7 days old rabbit. The increased expiratory flow is preceded by an increased tidal volume in the cough—but not expiration reflex.

The defensive responses triggered by mechanical stimulation of the trachea were identified from inspecting the respiratory cycle undergoing stimulation (stimulation breath) for a change in the tidal volume (VT) and peak expiratory flow (V'Epeak) from the preceding respiratory cycles (reference breath) and by the occurrence of abdominal muscle electromyographic (EMG) activity (Varechova et al., 2012). Cough reflex was defined as an increase of VT followed by an increased V'Epeak associated with abdominal contraction. Expiration reflex was defined as a brief V'Epeak of variable amplitude associated with an abdominal contraction, without prior increase in VT (Figure 2). In order to take into account the spontaneous between-breath variability, an unbiased differentiation of cough from expiration reflex was achieved by a statistical evaluation of VT between stimulation and reference breath. Tidal volume of reference breath was determined as the mean of 3 breaths prior to stimulation and its upper limit as mean + 3 standard deviations. The cough reflex was identified when VT of stimulation breath was higher than upper limit of reference VT. Occasionally, multiple defensive responses were elicited, consisting of a bout of several cough reflexes and/or expiration reflexes. Multiple responses were classified as cough reflex whenever such motor pattern was present in the bout.

#### Incidence of defensive responses

In each animal, the incidence of defensive responses (cough reflex and/or expiration reflex) elicited with 600 ms stimulus was expressed as percentage of the total number of stimulations. The frequency of cough reflex motor pattern in each animal was expressed as a percentage of all defensive responses elicited with 600 ms stimulus.

#### Sensitivity of defensive responses

In each animal, the threshold was defined as the shortest stimulus necessary to evoke at least one defensive response. When no response occurred using 2400 ms (longest) stimulation, the threshold was arbitrarily set to 4800 ms.

### Data analysis

Statistical analysis was performed using SYSTAT 13 Package (San Jose, CA, USA). Normal distribution of data within each age group was tested using Kolmogorov-Smirnov test. Age and weight data were normally distributed and are expressed as mean (95%CI). Defensive reflex incidence and threshold data are expressed as median (25th to 75th percentile) owing to non-normal distribution even after logarithmic transformation. The non-parametric Kruskal-Wallis test (a one-way analysis of variance) was used to measure differences in incidence and threshold data across the three feeding behavior groups. Pairwise comparisons between groups were subsequently performed using *post-hoc* analysis (Conover-Inman test for multiple comparisons) and a statistical significance was retained for a *p* ≤ 0.05.

## Results

Overall, 61 pups underwent mechanical stimulation of the trachea. Information on age and weight of rabbits in each group is detailed in Table 1.

**Table 1:** **Characteristics of studied rabbits**.

**Group**	**Suckling (<21 days)**	**Weanling (21–40 days)**	**Weaning (>40 days)**
*N*	22	21	18
Age (days)	13 (11–15)	31 (28–33)	79 (72–86)
Weight (grams)	244 (209–280)	726 (631–821)	2687 (2458–2916)

*Data are mean (95% confidence interval)*.

### Incidence of defensive responses at 600 ms

**Figure 3:**
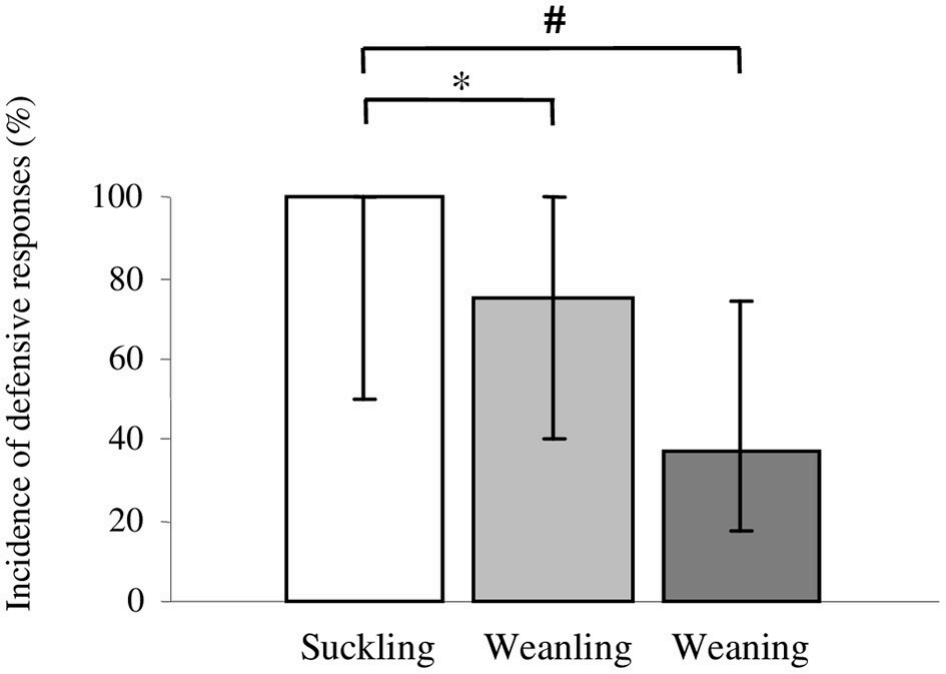
**Incidence of defensive responses elicited with 600 ms stimulus in the group of suckling (<21 days old), weanling (21–40 days old) and weaning (>40 days old) rabbits**. Pairwise comparisons between groups were provided by the non-parametric Kruskal-Wallis test using Conover-Inman *post-hoc* analysis. Values are median (25th to 75th percentile), ^*^p = 0.01; ^#^*p* = 0.05.

The incidence of defensive responses elicited using 600 ms stimulus was found to be significantly higher in the suckling [100% (50–100%); *p* = 0.01] and weanling [75% (40–100%); *p* = 0.05] group as compared to weaning group [37.5% (0–75%); Figure 3]. No significant difference in incidence of defensive responses was found between weanling and weaning group.

**Figure 4:**
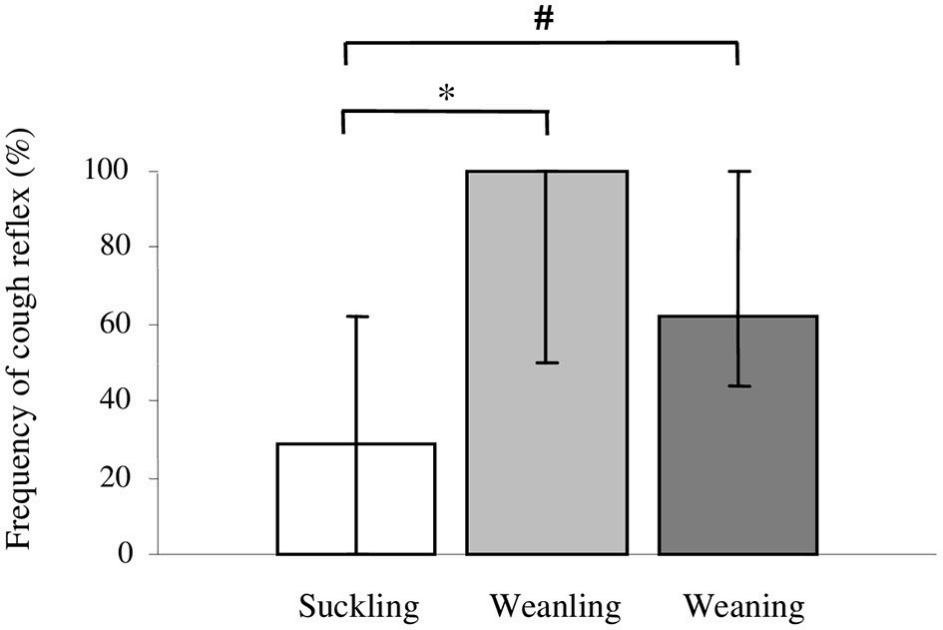
**Frequency of cough reflex motor pattern, expressed as a percentage of all defensive responses elicited with 600 ms stimulus in the group of suckling (<21 days old), weanling (21–40 days old) and weaning (>40 days old) rabbits**. Pairwise comparisons between groups were provided by the non-parametric Kruskal-Wallis test using Conover-Inman *post-hoc* analysis. Values are median (25th to 75th percentile), ^*^p = 0.006; ^#^*p* = 0.05.

The cough reflex accounted for 29% (0–62%) of all defensive responses elicited in the suckling group and its incidence was significantly lower when compared with weanling [100% (50–100%); *p* = 0.006] and weaning group [62% (50–100%), *p* = 0.05] (Figure 4).

### Sensitivity to mechanical stimulation

**Figure 5:**
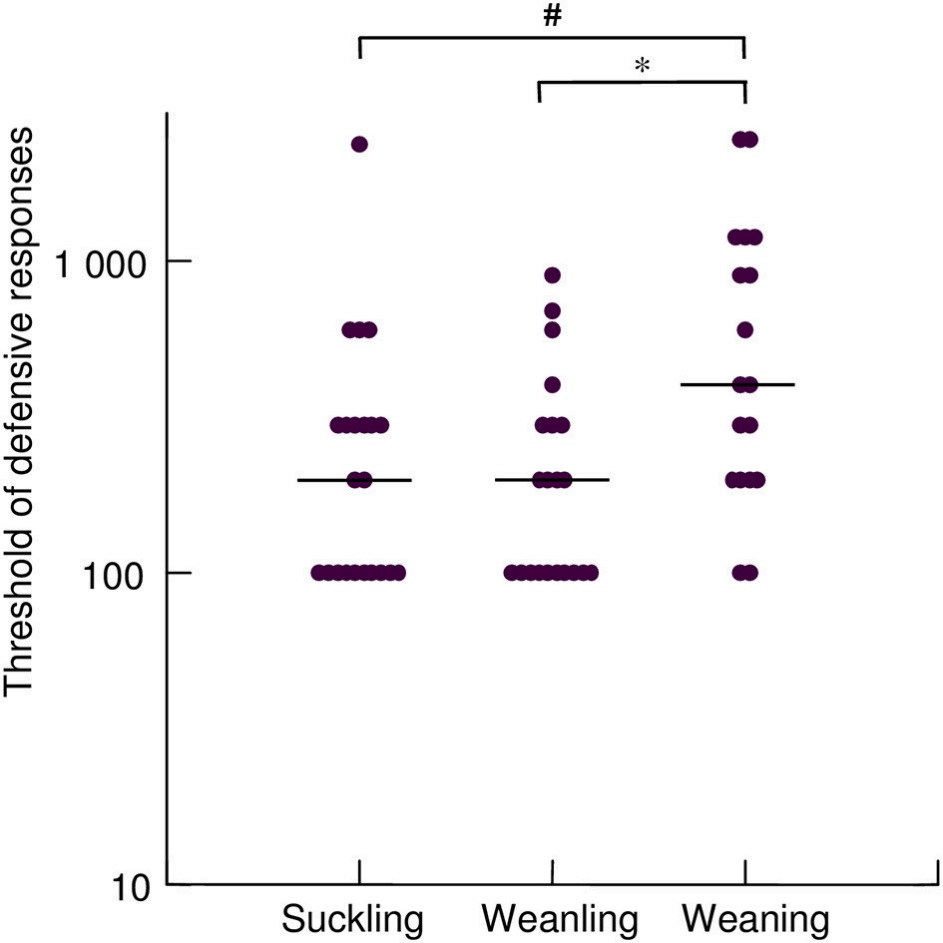
**Threshold of defensive responses (ms) defined as the shortest stimulus necessary to evoke at least one defensive response in the group of suckling (<21 days old), weanling (21–40 days old) and weaning (>40 days old) rabbits**. Pairwise comparisons between groups were provided by the non-parametric Kruskal-Wallis test using Conover-Inman *post-hoc* analysis. The filled circles are individual threshold values and full line indicates median values for each group. ^*^p = 0.003; ^#^*p* = 0.008.

The threshold of defensive response was significantly higher in the weaning group [400 ms (200–1200 ms)] as compared to suckling [200 ms (100–300 ms), *p* = 0.008] and to weanling ones [200 ms (100–300 ms); *p* = 0.003; Figure 5]. No significant difference in threshold was found between suckling and weanling group.

## Discussion

To the best of our knowledge, this is the first preclinical investigation of the maturation of airway defense reflexes elicited by a standardized mechanical stimulation of the trachea. Suckling pups showed increased incidence of defensive responses and lower stimulus threshold compared to more mature animals. The major type of response in those younger pups was the expiration reflex, while cough was more readily triggered in weaning animals.

An European Respiratory Society task force recommended a simple and unique definition for cough based on recognition of the characteristic sound, mainly for the clinical purpose of identifying symptom or disease (Morice et al., 2004). The usefulness of a more precise definition is defended from a physiological point of view (Chung et al., 2009), especially in the context of studying maturation, where a distinction between cough and expiration reflex appears essential. The cough reflex relies on the complex synchronization of a number of respiratory muscles to achieve a deep inhalation followed by glottis closure meant to pressurize the airways which helps generating near maximal expiratory airflow (Fontana and Lavorini, 2006). Experimental studies have shown the ability of laryngeal and tracheal stimulation to elicit such response (Sullivan et al., 1978; Tatar et al., 1994). The expiration reflex is a brief expiratory effort which—under experimental conditions - is mostly reported in response to laryngeal stimulation and may be triggered at any time of the breathing cycle, although it is most readily observed in expiration (Varechova et al., 2010). Cough has the primary role of cleaning the bronchial airways, eliminating excessive mucus production or incidentally inhaled foreign material. The expiration reflex is meant to prevent or limit inhalation of foreign matter presenting at the laryngeal opening (Korpas and Tomori, 1979).

In the observational study by Miller et al, a spontaneous cough was heard in the delivery room in 47% of unselected newborns (Miller et al., 1952). Experimental approaches in babies stimulated the pharyngeal area using mechanical or chemical stimuli (Thach, 2007). The external recording of breathing allowed identifying apnea and swallowing as major responses to chemical stimulation by distilled water or normal saline (Perkett and Vaughan, 1982; Pickens et al., 1988; Thach, 2007). Cough was occasional in immature subjects and became a more regular response with maturation (Thach, 2007). An analysis of infant responses to laryngeal stimulation under laryngoscopy reported that the frequency of the triggered vocal cord adduction, that was consistent with a cough - or an expiration - reflex increased from a few days to 1 month of age. Moreover, in that study where chemical and mechanical stimulations were tested, the incidence of responses was usually concordant with both stimuli (Miller et al., 1952).

The availability of animal models allowed more specific and systematic approaches to describe types of airway defense mechanisms to nociceptive stimuli applied to different parts of the airways. These preparations have made it possible to study not only pharyngeal and laryngeal stimuli but also those stimuli applied to the tracheo-bronchial tree which has received much less attention during development. An analysis of responses to upper airway stimulation was provided in the study by Korpas and Tomori (1979) in kittens. Both expiration and cough reflexes to laryngeal stimulation were readily observed by day 2, in contrast with the tracheo-bronchial stimulation that failed to regularly elicit a cough during the first week of life (Korpas and Tomori, 1979). In keeping with this pioneering experiment in kittens, the current results demonstrate that the cough reflex is hardly evoked by mechanical stimulation of the trachea in suckling rabbit pups, in contrast with the expiration reflex that is identified as the primary response, also more frequently occurring than in weaning animals. Moreover, suckling rabbits were found more sensitive to the mechanical stimulus compared with weaning animals. It is possible that the age related differential incidence of expiration and cough reflexes involves the maturation of lung mechanoreceptors in early development (Mortola and Fisher, 1988). Indeed, the number of rapidly adapting receptors that may be recorded from the dog (Fisher and Sant'Ambrogio, 1982) or opossum (Farber et al., 1984) vagus nerve has been suggested to be lower in pups than in adults, with the corresponding afferent volley eliciting a different response from the central neuronal network.

Maturation of defensive reflexes of the proximal airway is likely to be linked to important functions related to feeding, such as swallowing. Lau and coworkers (Lau and Schanler, 1996; Lau et al., 2003; Lau, 2015) emphasize the importance of the swallow-breathing interaction that must be coordinated at birth. The feeding behavior is successful when those rhythmic activities are in perfect synchrony, which implies coordination of oral, pharyngeal and laryngeal muscles. A number of upper airway muscle groups are similarly activated during swallowing and breathing but are coordinated by different central pattern generators (Broussard and Altschuler, 2000; Altschuler, 2001). A systematic analysis of these motor events in term newborns during bottle feeding showed that although swallowing may be triggered during a brief apnea, it may - in many instances—be observed to occur from any part of the breathing cycle (Lau et al., 2003). The synchronization of breathing and feeding activities has been studied in detail using combined functional and imaging methodologies in young infants. The glottis closure was identified as the first response triggered by a swallow, either occurring spontaneously, or provoked by pharyngeal infusion (Jadcherla et al., 2009). In the experimental conditions, the reflex glottis closure was a constant response reflecting the “hypervigilant state”—characteristic of the newborn - that is meant to protect the lower airways during liquid feeding (Jadcherla et al., 2009). Similar methodological approach in the newborn also identified a glottis closure in response to liquid infusion into the esophagus, although the reflex was sometimes absent (Jadcherla et al., 2007). Interestingly, when the occurrence of either the pharyngo-glottal reflex or the oesophago-glottal reflex is documented during breathing, either response may be found to be delayed. For instance, the stimulus applied in inspiration may be followed by glottal closure in expiration. Also, during bottle feeding a bolus transfer delay is necessary from the expression phase of suction to the pharyngeal phase of swallowing (Lau et al., 2003). As a result of any of these circumstances, a time lag exists where the oral-pharyngeal cavity is not cleared and therefore during which the lower airways may be at risk for aspiration. In this context, a fast expulsive effort provoked from trachea, preceded by glottis closure but not requiring a prior deep inhalation would be of particular benefit to eliminate the foreign material, and prevent further entry down into the bronchi. It is worth mentioning that the sensitivity to mechanical stimuli was found to be enhanced in the more immature animals (Figure 5).

In weanling animals, a different strategy for airway protection may become necessary with introduction of solid food. It is tempting to speculate that, because of food particles of larger size and of gravity, expulsion from the lower airways requires stronger expulsive effort. A deep preparatory inspiration of cough reflex, serving to optimize the pre-contractile lengths of the expiratory muscles, is important to facilitate dynamic airway compression and to generate near maximal expiratory air velocities during the expulsive phase of cough (Fontana, 2003). As synchronization between relatively fast events such as sucking, swallowing and respiration is no longer necessary, the breathing cycle may be interrupted for the longer period of time necessary to promote deep breath and lung volume compression. With further maturation, in weaning animals, the threshold to mechanical stimulation of the trachea increases. To the extent that maturational processes may compare between humans and rabbits at different stages of development, it is interesting to note that the current findings are in keeping with the prior report of a decreased sensitivity to capsaicin challenge tests in teenagers compared with younger children (Varechova et al., 2008). Also the current animal study is in agreement with the clinical observation that Pertussis in young children manifests itself more frequently with the characteristic whooping cough than in adults (Chang, 2005; Widdicombe and Fontana, 2006).

It is concluded that the airway defense mechanisms involved in ventilatory responses to mechanical stimulation of the trachea undergo considerable change during maturation. Suckling rabbit pups were found more sensitive to this stimulus than weaning animals, also exhibiting more frequent responses in the form of the expiration reflex rather than cough, a pattern that appears quite suited to their feeding behavior. The frequency of cough on the other hand appears more frequent in older animals, where feeding solid food occurs.

## Author contributions

LC, II, SD, CS, and FM have prepared the project of this study. LC, SD, BD, LD, and AL managed preparatory phase of the study. LC, SD, BD, LD, and AL performed measurements and assured technical assistance in the laboratory. LC, II, LD, SD, and CB performed data collection and statistics. LC, II, SD, and FM have prepared the draft of manuscript. LC, II, SD, BD, CB, CS and FM completed the work and revised the final manuscript.

### Conflict of interest statement

The authors declare that the research was conducted in the absence of any commercial or financial relationships that could be construed as a potential conflict of interest.
